# The Dual Role of Glial Extracellular Vesicles in Neurodegeneration: Insights from iPSC-Based Models

**DOI:** 10.3390/ijms27125182

**Published:** 2026-06-08

**Authors:** Aurora Scrivo, Liliana Bernardino, Antonella Consiglio

**Affiliations:** 1 Department of Pathology and Experimental Therapeutics, Bellvitge University Hospital IDIBELL, Hospitalet de Llobregat, 08908 Barcelona, Spain; 2 RISE-Health, UBI, Faculty of Health Sciences, University of Beira Interior, 6200-506 Covilhã, Portugal; libernardino@fcsaude.ubi.pt; 3 Institute of Biomedicine of the University of Barcelona (IBUB), 08028 Barcelona, Spain

**Keywords:** extracellular vesicles, iPSC, glial cells, neurodegenerative disease

## Abstract

Extracellular vesicles (EVs) have emerged as key mediators of intercellular communication in the brain, with glial cell-derived EVs increasingly recognized for their roles in maintaining brain homeostasis and contributing to the progression of neurodegenerative diseases. By transferring a diverse cargo of bioactive molecules, including proteins, RNAs, and organelles, EVs influence recipient cell behavior and overall brain function. In neurodegenerative conditions, glial EVs can either propagate pathogenic signals or deliver neuroprotective and regenerative cues, depending on their cellular origin and molecular composition. This context-dependent heterogeneity highlights the need for physiologically relevant human models to investigate EVs biology. Human induced pluripotent stem cell (iPSC)-derived glial models provide a disease-relevant platform, as they recapitulate key pathological features of Alzheimer’s disease (AD), Parkinson’s disease (PD) and amyotrophic lateral sclerosis (ALS). When further integrated with brain organoid platforms, these iPSC-based systems enable the generation of three-dimensional environments that closely resemble in vivo EVs dynamics. Importantly, glial EVs can modulate cellular pathways involved in neuronal survival and function. Indeed, their potential to interact with and, under specific experimental conditions, traverse the blood–brain barrier (BBB) has contributed to growing interest in their application for biomarker discovery and therapeutic development. Engineered and patient-specific EVs derived from iPSCs are emerging as promising tools for targeted, cell type-specific, therapeutic approaches, although their clinical applicability still requires further validation. This review discusses the emerging evidence supporting the dual role of iPSC-derived glial EVs in health and disease, underscores the translational potential of iPSC-based platforms for mechanistic studies, and outlines their promise as precision medicine tools for diagnostics and therapy.

## 1. Introduction

Neurodegenerative diseases (ND) represent a major subgroup of neurological disorders and are characterized by the progressive and selective loss of specific neuronal populations, leading to irreversible cognitive, motor, and behavioral impairments. Despite their clinical heterogeneity, conditions such as Alzheimer’s disease (AD), Parkinson’s disease (PD), and amyotrophic lateral sclerosis (ALS) share common pathogenic mechanisms, including protein aggregation, neuroinflammation, and widespread disruption of intercellular communication within the central nervous system (CNS). More specifically, altered intercellular communication has been independently reported in AD [1], ALS [2], and PD [3], together with disruptions in intracellular mechanisms such as oxidative stress and mitochondrial dysfunction [4,5,6]. Furthermore, a shared genetic architecture has been identified across AD, ALS, Lewy body dementia and PD, highlighting common loci (TMEM175 and HLA), genes, pathways, and tissues implicated in multiple neurodegenerative disorders [7].Together, these alterations profoundly reshape the brain microenvironment and compromise global neural network functionality [8,9].

In the context of ND, a major advance has been the development of induced pluripotent stem cells (iPSCs), first described by Shinya Yamanaka and colleagues in 2006. This technology relies on the reprogramming of human somatic cells, such as fibroblasts, through the ectopic expression of defined transcription factors, thereby restoring pluripotency [10]. The use of iPSCs provides a decisive advantage for ND research, as it enables the generation of patient-specific neural and glial cells that retain the donor’s genetic background. iPSC-derived glial cells express human-specific genes, splice variants, and regulatory elements, more accurately reflecting human central nervous system biology than rodent models. Moreover, iPSCs can be established from patients carrying defined pathogenic mutations (e.g., familial AD, ALS, or PD) or genetic risk alleles, allowing the investigation of disease-specific glial phenotypes and molecular signatures [11,12,13]. To illustrate this concept, iPSC-derived glial models have revealed disease-specific effects of distinct splice variants and regulatory pathways in neurodegeneration-related genes. For example, PD-associated *TMEM175* variants in iPSC-derived astrocytes and microglia alter the balance between full-length and lysosomal-targeted splice isoforms, impairing lysosomal pH regulation and α-synuclein (α-syn) clearance through NF-κB/inflammasome-associated inflammatory pathways [14]. Similarly, familial ALS-linked *SOD1* mutations in iPSC-derived astrocytes affect the relative abundance of cytoplasmic versus mitochondrial-targeted splice isoforms, leading to dysregulated Nrf2–KEAP1 and NF-κB signaling, oxidative stress and non-cell-autonomous motor-neuron toxicity [15]. Importantly, human iPSC-derived microglia and astrocytes display stimulus-dependent cytokine and chemokine responses that closely resemble those observed in human tissue [16,17].

Despite substantial advances in elucidating the mechanisms underlying ND onset and progression, an urgent need remains to identify early biomarkers and to comprehensively characterize the altered molecular pathways, ultimately paving the way for effective disease-modifying therapies. Increasing attention is placed on the role of cell-cell communication, and specifically glia-neuron communication. Accumulating evidence over the past two decades has repositioned glial cells as active drivers of ND onset and progression, rather than passive bystanders [18,19].

Glial cells regulate brain homeostasis and pathology in part through the release of secretory factors, which can be either freely secreted or packaged within extracellular vesicles (EVs). Initially considered a cellular waste disposal mechanism, EVs are now recognized as key mediators of intercellular communication. Numerous studies have demonstrated that EVs are efficiently taken up by neighboring or distant cells, where they actively modulate cellular behavior. By transferring bioactive cargo, including proteins, lipids, and nucleic acids, EVs act as important signaling entities involved in the regulation of physiological and pathological processes [20].

Recent transcriptomic and proteomic studies are starting to characterize EVs cargo released by iPSC-derived glia, linking specific EVs components to neuroprotective pathways, including PI3K–Akt activation, anti-apoptotic signaling, and synaptotrophic programs [21]. Notably, glial EVs from healthy donor iPSCs improve cognitive deficits and reduce neuropathology in mouse models, highlighting their therapeutic potential.

In the context of ND, EVs are particularly relevant because they can export stress-associated cargo when degradative pathways are challenged and by that contributing to the propagation of the pathology and the amplification of inflammatory programs driven by glial cells.

In this review, we aim to examine human iPSC-derived glial EVs as both mechanistic drivers and translational substrates in ND, with emphasis on their potential as cell-type-specific biomarkers and therapeutic targets. We highlight how their effects range from propagating pathology to supporting protection and repair depending on glial subtype, activation state, and cargo composition. We also discuss how iPSC-based models and integration into organoids enable human-relevant dissection of EVs biology and accelerate the development of EVs-based therapeutic strategies.

### 1.1. EVs Biology

EVs represent a heterogeneous group of membrane-bound structures released in the extracellular space by potentially all cell types. They originate either from the endosomal system or directly from the plasma membrane and are commonly classified based on their size and biogenesis. Although the field still lacks robust standardization of EVs subpopulations, EVs are broadly categorized into three main classes: exosomes, microvesicles, and apoptotic bodies [22]. Exosomes, typically ranging from 50 to 150 nm in diameter, originate from the endosomal pathway. They are generated through inward budding of the endosomal membrane, leading to the formation of intraluminal vesicles (ILVs) within multivesicular bodies (MVBs). Upon fusion of MVBs with the plasma membrane, ILVs are released into the extracellular space as exosomes through a tightly regulated process involving multiple molecular components. Exosomes are typically enriched in tetraspanins such as CD63 and CD81, along with endosomal markers like Alix and TSG101, which reflect their origin from MVB [23]. Microvesicles are larger vesicles (approximately 150 to >1000 nm) formed through direct outward budding and fission of the plasma membrane and are characterized by a distinct lipid composition. These vesicles can transport diverse biomolecules, including ribosomal RNA (rRNA) and messenger RNA (mRNA). They often carry cell-type–specific receptors and adhesion molecules (e.g., selectins or integrins) as well as flotillin-2, associated with plasma-membrane-derived vesicles [24]. Apoptotic bodies, the largest EVs subtype (1–5 µm), are generated during programmed cell death through membrane blebbing and cellular fragmentation. They are characterized by abundant surface-exposed phosphatidylserine (PS), along with histone fragments and complement-related proteins such as C3b and C1q, reflecting their derivation from fragmented apoptotic cells that facilitate their recognition and clearance by phagocytic cells, such as macrophages [25,26]. The relative abundance and molecular composition of EVs differs between neurons and glial cells, and both are dynamically regulated by physiological state and disease. Neurons predominantly release small EVs enriched in synaptic and membrane-associated proteins which are linked to processes like synaptic plasticity and axonal communication [27,28]. In contrast glial cells, including astrocytes, oligodendrocytes and microglia, produce a broader spectrum of vesicles ranging from exosomes to larger microvesicles, with microglia in particular increasing microvesicle release during inflammatory activation and packaging pro-inflammatory mediators [29]. Under homeostatic conditions, astrocytes tend to release more exosome-associated vesicles, whereas pathological stimuli such as neuroinflammation, oxidative stress, or amyloid-β exposure shift glial populations toward increased microvesicle shedding [30,31]. Accumulating evidences indicates that EVs can also originate from additional intracellular compartments, including mitochondria [32], secretory lysosomes and amphisomes [33,34]. Remarkably, EVs and EV-associated structures have been reported to transport mitochondrial components and, in some contexts, mitochondria-containing vesicles capable of influencing metabolic activity in recipient cells [35]. However, the mechanisms, reproducibility, and physiological relevance of these observations remain incompletely understood and continue to be actively investigated. This added layer of complexity further complicates the precise definition and classification of EVs subpopulations. Because EVs classes overlap in size and composition, rigorous interpretation increasingly relies on standardized isolation and on cell-type-resolved marker strategies rather than size alone. Once released into the extracellular space, EVs can be internalized by recipient cells through multiple mechanisms, including direct fusion with the plasma membrane, clathrin-dependent and clathrin-independent endocytosis, and micropinocytosis [36,37]. Beyond these general uptake pathways, increasing evidence suggests that EVs can exhibit cell-type specificity. For instance, exosomes derived from neuroblastoma cells were found to bind both neurons and glial cells but to be preferentially endocytosed by glia. Alternatively, exosomes released from stimulated cortical neurons were taken up exclusively by neurons, indicating that activity-dependent neuronal exosomes exhibit selective homotypic targeting and may represent a mechanism for specialized interneuronal communication [38]. Consistent with the idea of EVs cell-type specific effect, microglial EVs show stimulus and recipient-dependent specificity. In fact, pro-inflammatory EVs impair oligodendrocytes progenitor cells (OPC) maturation in an astrocyte-dependent manner, while pro-regenerative EVs enhance OPC recruitment and promote remyelination, underscoring highly selective EVs effects on distinct neural cell populations during myelin repair [39]. These studies highlight a preferential uptake of EVs by defined target cells, indicating that EVs release is a highly regulated and context-dependent process with selective molecular cargo tailored to modulate specific signaling pathways [40,41].

### 1.2. Interplay Between Lysosomal Degradation Pathways and EVs Secretion

**Figure 1:**
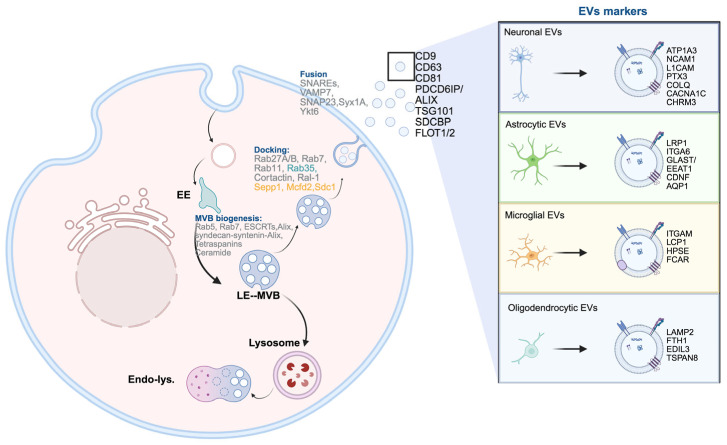
EVs biogenesis and cell-specific EVs markers. The figure represents the biogenesis of EVs at the interface between degradation and secretion and highlights the molecular regulators involved in this phase, specifically MVBs biogenesis, docking and fusion. Intracellular general regulators are presented in gray, cell-specific regulators are presented in a different color (blue: neurons; bright green: astrocytes; orange: microglia; sea blue: oligodendrocytes). The panel on the left include cell-specific EV marker. This figure was created using BioRender: https://BioRender.com/apdbutd, accessed on 24 May 2026.

Due to both origin and function, EVs are closely connected to cellular degradation systems, particularly the endo-lysosomal pathway, in which lysosomes act as terminal organelles responsible for the breakdown of cellular components through hydrolytic enzymes. This relationship is inherently dynamic, as degradative and secretory pathways share multiple intracellular compartments. MVBs represent a key decision point, as they can either fuse with lysosomes for cargo degradation or with the plasma membrane to release ILV as exosomes [42] (Figure 1). Although the molecular mechanisms governing this fate choice are not fully understood, several regulatory components have been identified.

EVs are enriched in tetraspanins (CD63, CD9, CD81), commonly used as EVs markers and implicated in ILV formation [43]. Specific regulators, such as tetraspanin 6, can influence the balance between degradation and secretion [44]. The Endosomal Sorting Complex Required for Transport (ESCRT) machinery plays a central role in cargo sorting within MVBs, while small GTPases of the RAB family regulate MVBs trafficking, either promoting fusion with the plasma membrane or directing maturation toward lysosomal degradation. Importantly, the activity of these regulators is often cell type-specific. For example, RAB35 has been shown to control EVs secretion in oligodendrocytes, and constitutively active RAB35 in neurons enhances extracellular release of proteins, including α-synuclein, supporting a role in EVs-mediated protein propagation [45,46]. Interestingly, Rab35 has been previously identified as a key regulator of α-syn seed transfer and propagation. Mechanistically it has been demonstrated that it mediates the contact-dependent spread of α-syn fibrils from enteroendocrine to neuronal cells via endocytic trafficking, while its inhibition promotes lysosomal clearance and reduces pathological transmission [47]. Cell type-dependent regulation of EVs secretion has also been described in microglia, where specific regulators such as Sepp1, Mcfd2, and Sdc1 have been identified [48,49]. A detailed overview of cell type-specific mechanisms in brain cells is provided in Figure 1.

The functional link between degradation and secretion is further highlighted by shared molecular regulators. LC3, a key component of the autophagy machinery, participates in cargo loading and redirects RNA-binding proteins into MVBs for secretion [50]. In addition, reactive astrocytes upregulate mTOR, a major regulator of the endo-lysosomal pathway, and release pro-inflammatory cytokines such as IL-32 via EVs, illustrating how EVs content dynamically reflects cellular activation states [51]. Conversely, impairment of lysosomal function shifts the balance from degradation toward secretion [52]. Chemical or genetic inhibition of lysosomal activity increases the release of both small and large EVs containing autophagy-related cargo, including mitochondrial components that would normally be degraded.

**Figure 2:**
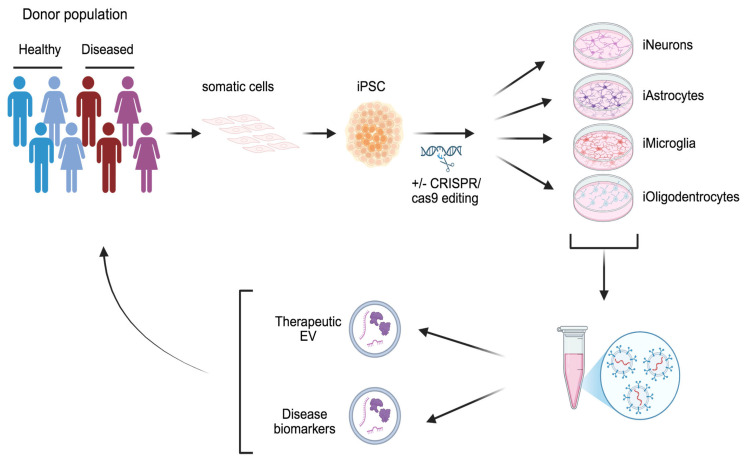
iPSCs as a model to study EVs. iPSCs can be generated from somatic cells obtained from healthy individuals or affected patients. iPSCs can be genetically modified using CRISPR/Cas9 to introduce or correct disease−relevant mutations, enabling the creation of isogenic control lines. By applying specific differentiation factors, iPSCs derived from the same individual can be directed into various brain cell types, from which EVs can be isolated and characterized. Analysis of EVs molecular signatures, whether from patient−derived, healthy, or isogenic lines, provides insights that can be translated back to the clinical setting, supporting a comprehensive bench−to−bedside approach. This figure was created using BioRender: https://BioRender.com/ebpvxgn, accessed on 24 May 2026.

Under pathological conditions, this compensatory secretory response may contribute to disease progression by promoting the extracellular spread of aggregation-prone proteins [53]. The biological impact of EVs largely depends on their molecular cargo. In NDs, three major cargo categories appear particularly relevant: (i) aggregation-prone proteins, such as α-syn, which can promote intercellular seeding and pathological spreading [54,55,56]; (ii) organellar and autophagy-related components, reflecting impaired degradative capacity [57]; and (iii) RNA and RNA-binding protein modules capable of altering transcriptional and translational programs in recipient cells [58,59]. Interestingly, prior alteration such as lysosomal dysfunction may lead to divergent secondary effects depending on EVs composition and the identity or vulnerability of target cells. This framework suggests that restoring lysosomal flux could reduce EVs release and partially normalize EVs cargo, providing a mechanistic link between proteostasis and EVs-mediated intercellular signaling in ND, although further mechanistic studies are required. In this context, iPSC-derived cells represent a powerful platform to investigate EVs biology. EVs generated from iPSC-derived neurons, astrocytes, microglia, and oligodendrocytes display distinct cell type-specific protein signatures (Figure 1), supporting the concept of EVs heterogeneity based on cellular origin and allowing the discovery of cell type-specific biomarkers [60]. Neuron-derived EVs are enriched in synaptic and neuronal proteins, including SYT1, STX1B, NCAM1, RTN1, and ATP1A3. Microglia-derived EVs display immune-related signatures characterized by ITGAM (CD11b), ITGB2, CD300A, and LCP1, consistent with phagocytic and inflammatory functions. Astrocyte-derived EVs are enriched in proteins associated with extracellular matrix organization, metabolism, and lipid transport, including APOE, LRP1, ITGA6, SLC transporters, VIM, and GFAP-linked pathways. Oligodendrocyte-derived EVs show enrichment of myelin- and lysosome-related proteins such as PLP1, MBP, CNP, LAMP2, and FTH1. Together, these EV proteomic profiles provide robust cell type-specific signatures that support EV-based cell-of-origin tracing in the brain [60]. Moreover, the EVs proteome may serve as a functional readout of the cellular degradative state and allow the association between genetic background, cellular stress, lysosomal function and EVs cargo composition. Indeed, impaired autophagy in neurons carrying the PD–associated LRRK2G2019S mutation, leads to rerouting of autophagic intermediates toward the secretory pathway, resulting in increased EVs release enriched in autophagic and mitochondrial proteins, reflecting a compensatory mechanism for defective lysosomal degradation [57]. In line with this concept, studies in *Caenorhabditis elegans* have shown that proteostatically stressed neurons (such as impaired chaperone function, autophagy, proteasome activity, or mitochondrial dysfunction) can extrude large membrane-bound vesicles (exosphers) containing protein aggregates and damaged organelles, thereby highlighting a conserved mechanism of neuronal proteostasis and organelle quality control [61]. Beyond biomarker discovery, iPSC-based systems precise genetic manipulation and the generation of isogenic lines, allowing direct attribution of EVs alterations to disease-causing mutations. These models also provide a platform for identifying therapeutic strategies aimed at restoring lysosomal function and normalizing EVs-mediated signaling (Figure 2). Although only a limited number of clinical studies have explored the use of EVs to monitor or modulate brain disease progression, these findings highlight the strong translational potential of targeting the lysosome-EVs axis (Table 1).

### 1.3. Methodological Approaches for EVs Isolation and Characterization

A major challenge in EVs research remains the standardization of isolation, purification, fractionation, and storage methodologies, as differences in experimental procedures can substantially affect EVs output, purity, integrity, and molecular composition. Classical approaches such as differential ultracentrifugation (DUC) and density-gradient ultracentrifugation (DGUC) remain among the most widely used methods for EVs isolation. In particular, DGUC improves EVs purity by separating vesicles from lipoproteins and protein aggregates according to buoyant density. Recent studies have shown that combining size-exclusion chromatography (SEC) prior to DGUC (SEC-DGUC) improves RNA and protein recovery from small plasma volumes compared with the reverse workflow, while miniaturized fixed-angle rotor systems considerably shorten (up to 3 h) processing times while maintaining high-purity small EVs fractions [62]. SEC is considered one of the gentlest and most reliable EVs isolation methods, frequently integrated into hybrid workflows, including ultrafiltration-SEC pipelines to improve both EVs amount and purity, in particularly suitable for clinical and translational applications [63]. Fractionation strategies are also becoming increasingly important for resolving EVs heterogeneity. SEC-DGUC workflows have identified EVs-enriched high-density fractions (~≥1.08–1.10 g/mL) that can be clearly separated from lipoprotein-dominated fractions, whereas small-volume DGUC protocols enable the rapid generation of highly purified EVs suitable for downstream electron microscopy, western blotting, RNA analysis, and flow cytometry applications [62].

Ultrafiltration and tangential flow filtration (TFF) are increasingly used as scalable front-end approaches for large-volume processing, buffer exchange, and EVs concentration due to their high recovery efficiency and compatibility with downstream purification methods. In contrast, polymer-based precipitation approaches, such as polyethylene glycol (PEG)-mediated isolation, provide high yield and operational simplicity but frequently co-isolate lipoproteins and protein complexes [64]. Consequently, these methods are often combined with chromatographic purification steps to improve EVs specificity. More recently, multimodal and affinity chromatography approaches have emerged as powerful strategies for contaminant depletion and EVs subpopulation enrichment. Affinity-based capture methods using antibodies against canonical EVs markers, including CD9, CD63, and CD81, or phosphatidylserine-binding molecules, enable the selective isolation of defined EVs populations, although they may introduce marker-dependent selection bias and reduced throughput [63]. Importantly, hybrid purification pipelines combining TFF, PEG precipitation, and multimodal or affinity chromatography have demonstrated the feasibility of generating high-purity EVs preparations suitable for translational and GMP-oriented applications without requiring ultracentrifugation [65,66].

Microfluidic technologies are rapidly emerging as promising alternatives for EVs separation and characterization. Affinity-based microfluidic devices employ antibody- or aptamer-functionalized channels to selectively capture EVs subtypes with high specificity, although their performance may be limited by epitope heterogeneity and low scalability [67,68]. In parallel, label-free microfluidic systems separate EVs according to biophysical properties such as size, deformability, electric charge, acoustic contrast, or hydrodynamic behavior using approaches including acoustofluidics, inertial focusing and field-flow fractionation (FFF) [69]. Importantly, combining orthogonal separation principles including size, density, and affinity-based approaches, substantially improves EVs specificity and enables more efficient depletion of contaminants such as HDL, LDL, VLDL particles and ribonucleoprotein complexes [62,70].

Pre-analytical variables and storage conditions also critically influence EVs stability and reproducibility. Current MISEV2023 recommendations emphasize the importance of standardized workflows, minimal freeze-thaw cycles and include nuclease treatment to eliminate extravesicular nucleic acids. It result important to add orthogonal quality-control approaches including nanoparticle tracking analysis (NTA), electron microscopy, western blotting, RNA profiling, and functional uptake assays to ensure batch-to-batch consistency and long-term stability [26]. These technical considerations are particularly relevant for iPSC-derived EVs studies and for the future clinical translation of EVs-based biomarkers and therapeutic applications.

## 2. Role of iPSC-Derived Glial EVs in Cell-Cell Communication

Human iPSC-derived astrocytes, oligodendrocytes and microglia recapitulate key aspects of the human brain’s physiological and pathological environment, enabling investigation of the molecular signals that glial EVs convey to neurons and other cell types. This provides valuable insight into mechanisms of intercellular communication, neurodevelopment, and neurodegeneration [26]. Of note, iPSC-derived glial cells display heterogeneous and context-dependent responses to immune activation, reflecting their diverse roles in neuroinflammation [16].

Although the molecular characterization of glial-derived EVs—particularly those from iPSC-derived glial cells—remains limited, research in this area is rapidly expanding. Since glial cells actively respond to pathological stimuli, their EVs can carry signatures of cellular activation, inflammatory states and disease progression [71,72,73]. This is particularly relevant because several candidate biomarkers of neurodegeneration are preferentially expressed in non-neuronal cells.

**Figure 3:**
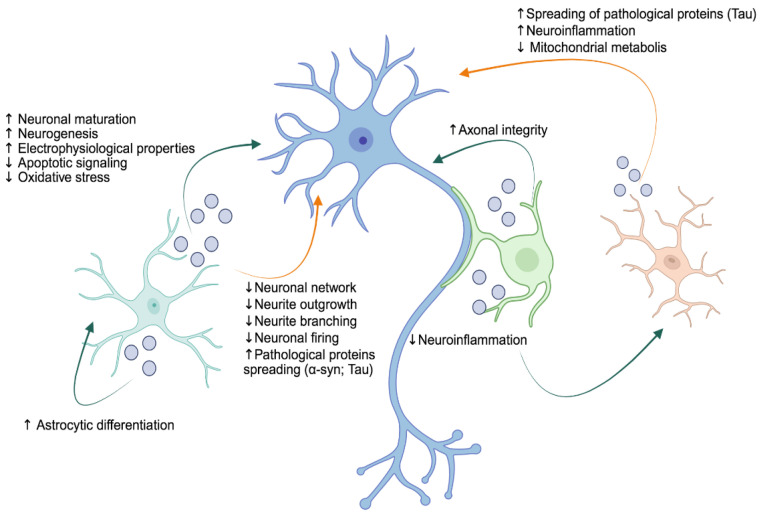
Physiological and pathological effect of iPSCs glia-derived EVs. EVs derived from glial cells (including astrocytes, microglia and oligodendrocytes), exert in the brain a variety of effects. The figure highlights the recent discoveries in the field using iPSC-derived cells, displaying both the beneficial (green arrow) and deleterious (orange arrow) outcome that have been reported. This figure was created using BioRender: https://BioRender.com/gmh938n, accessed on 24 May 2026.

Recent studies using iPSC-derived glial cells are beginning to clarify the roles of glial EVs in brain’s function and their contributions to both physiological and pathological processes, including AD, PD, and ALS (Figure 3). Collectively, these data support the idea that iPSC-derived glial EVs may represent a valuable and still understudied source of potential biomarkers. Together, these findings highlight the dual role of glial EVs in AD. Their cell-type specificity, detectability in peripheral biofluids, and sensitivity to disease-associated molecular changes support their potential to improve diagnosis, patient stratification and disease monitoring in neurodegenerative conditions.

In the following sections we summarize the recent discoveries on EVs, focusing on observations made from EVs derived from iPSC-based astrocytes, microglia and oligodendrocytes, highlighting their roles in brain function and their contribution to disease mechanisms underlying AD, PD, and ALS (Figure 3).

### 2.1. Astrocytes-Derived EVs

Astrocytes are specialized glial cells that play a central role in maintaining neuronal homeostasis by regulating synaptic activity, providing metabolic support, preserving blood–brain barrier (BBB) integrity, and modulating neuroinflammatory responses. In the healthy CNS, astrocyte-derived extracellular vesicles (ADEVs) contribute to neuronal function and brain homeostasis by delivering neurotrophic factors such as brain-derived neurotrophic factor (BDNF), glial cell line-derived neurotrophic factor (GDNF), and nerve growth factor (NGF) [74,75], as well as antioxidant enzymes including superoxide dismutase (SOD), catalase, glutathione-related enzymes and peroxiredoxins. ADEVs also transport metabolic intermediates such as lactate and molecules involved in mitochondrial support and redox balance promoting neuronal survival, synaptic activity and energy homeostasis [76,77]. ADEV’s protein cargo includes neuroprotective factors such as heat shock proteins, lipoprotein receptor-related protein 1 (LRP1), potassium channel tetramerization domain-containing 12 (KCTD12), glucose-6-phosphate dehydrogenase (G6PD), kinesin family member 5B (KIF5B), and spectrin-alpha non-erythrocytic 1 (SPTAN1). These proteins not only modulate neuronal differentiation but also support the acquisition of functional electrophysiological properties [78,79].

In addition to proteins, the RNA cargo of ADEVs regulates gene expression in recipient neurons, influencing synaptic plasticity, dendritic spine remodeling, and neurogenesis. For example, iPSC-derived astrocytes efficiently secrete EVs enriched in microRNAs (miRNAs) such as miR-21, miR-29a, miR-124, and miR-26a, which are involved in neuronal regulation and astrocyte identity [80,81,82,83,84].

More recently, ADEVs have been shown to support the neurovascular unit by transferring angiogenic and metabolic signals, such as VEGF, FGF2, ANGPT1 and pro-angiogenic miRNAs (e.g., miR-126) to endothelial cells, contributing to BBB integrity and neurovascular health [85]. These properties highlight the strong translational potential of ADEVs. iPSC-based models further provide a platform to investigate how different astrocytic states—resting versus reactive—affect EVs release, cargo composition, and functional outcomes in recipient cells. Continued study of iPSC-derived ADEVs will advance our understanding of glial biology while opening avenues for biomarker discovery and therapeutic development.

### 2.2. Microglia-Derived EVs

Microglia are the resident innate immune cells of the CNS, where they play a key role in maintaining tissue homeostasis by clearing the extracellular space and dynamically regulating neuroinflammatory processes. Microglia-derived extracellular vesicles (MDEVs) have historically received less attention, partly due to the relatively low abundance of microglia in the brain and the limited availability of robust tools for their specific isolation. However, recent advances in iPSC-based models and EVs isolation techniques are beginning to overcome these limitations.

MDEVs are gaining increasing interest for their potential roles in neuroinflammation, intercellular communication and the propagation of pathological proteins in ND. Due to technical limitations, most studies have focused on injury- and disease-related contexts. For instance MDEVs detected in plasma carry markers of microglial activation; following stroke, TMEM119^+^/CD14^+^ and TMEM119^+^/MHC-II^+^ EVs populations increase, reflecting pro-inflammatory and antigen-presenting phenotypes associated with disease progression [86].

The functional state of microglia is also shaped by interactions with other neural cells. iPSC-derived neural stem cells can modulate microglial polarization, suggesting that EV-mediated signaling contributes to regulating immune responses. Disruption of the balance between intracellular degradation and extracellular secretion is a key feature of microglial dysfunction in disease. In C9orf72-deficient models linked to ALS, impaired autophagy enhances EVs release alongside increased cytokine secretion, highlighting a connection between cellular stress and EV-mediated signaling [87].

Altogether, iPSC-derived microglia represent a physiologically relevant source of EVs and a valuable model for studying microglia-mediated communication and developing EV-based strategies in NDs.

### 2.3. Oligodendrocytes-Derived EVs

Oligodendrocytes are specialized glial cells responsible for axonal myelination and metabolic support, contributing to neuronal integrity and long-term circuit stability. The study of oligodendrocyte-derived extracellular vesicles (ODEVs) remains relatively limited, particularly for those released from iPSC-derived oligodendrocytes. Nevertheless, available evidence indicates that ODEVs carry specific factors that are essential for axonal maintenance and long-term neuronal support [88].

Beyond their physiological roles, ODEVs show therapeutic potential in immune-mediated disorders. Notably, they naturally contain multiple myelin antigens and have been shown to restore immune tolerance and reduce neuroinflammation in experimental models of multiple sclerosis, supporting antigen-specific therapeutic strategies without requiring prior identification of a single target antigen [89]. ODEVs are also increasingly recognized for their ability to promote remyelination. They transport myelin-associated proteins such as myelin basic protein (MBP), proteolipid protein (PLP) and myelin oligodendrocyte glycoprotein (MOG), as well as lipid components essential for myelin membrane assembly, including sphingolipids and cholesterol. In addition, they carry metabolic enzymes supporting axonal energy homeostasis and regulatory RNAs, such as miR-219 and miR-338, which are involved in oligodendrocyte differentiation and myelin gene expression [90]. ODEVs have been implicated in neuroinflammatory and demyelinating conditions, where they contribute to immunomodulation and axonal support [91], although direct evidence in tauopathies remains limited. Ruan and colleagues reported increased enrichment of myelin oligodendrocyte glycoprotein (MOG), a mature oligodendrocyte marker, in AD’s EVs samples compared to preclinical AD and control samples, indicating altered oligodendrocyte-associated EV signaling in neurodegeneration [92]. Additionally, independent proteomic analyses have detected 10–40% of known brain cell type marker proteins across EV fractions, with oligodendrocyte, glutamatergic neuron and astrocyte markers showing the highest enrichment [93].

These vesicles may act not only as carriers of regenerative signals to damaged axons but also as modulators of the local immune microenvironment, supporting tissue repair and functional recovery. Although the field is still in its early stages, iPSC-derived ODEVs represent a promising platform for regenerative and immunomodulatory approaches. Further characterization of their molecular cargo, mechanisms of action and translational feasibility will be essential to fully harness their potential for myelin repair and neuroprotection.

## 3. iPSC-Derived Glial EVs in Neurodegeneration

Glial EVs are increasingly recognized as important mediators of neurodegenerative processes, acting as vehicles that transfer inflammatory signals, misfolded proteins, and regulatory RNAs between microglia, astrocytes and neurons (Table 2). Recent work using iPSC-derived glial models has highlighted that glia-derived EVs function as dynamic mediators of glia–neuron communication whose biological impact depends critically on the activation state of the parental glial cells and the underlying disease context. In AD and related neuroinflammatory diseases, EVs from iPSC-derived neural stem cells or microglia can carry anti-inflammatory and neuroprotective cargo that reduces microglial activation, astrogliosis and amyloid-associated pathology in vivo [94,95]. By contrast, EVs released by reactive or disease-activated glia, such as HIV-1 Tat-exposed astrocytes, often carry neuropathogenic factors that promote neuroinflammation and neuronal dysfunction [96]. In support of this idea, investigations using patient-derived iPSCs glial cells indicate that disease-associated transcriptional reprogramming and chronic immune activation can modify EVs composition toward a more detrimental profile, potentially enhancing antigen presentation pathways and propagating neurotoxic signals in disorders such as PD and multiple system atrophy [97]. Complementing these observations, more recent studies have reported that EVs released from human iPSC-derived glial progenitor cells can protect neurons from glutamate-induced excitotoxicity by reducing intracellular calcium levels, preserving mitochondrial membrane potential and activating pro-survival pathways such as PI3K-Akt signaling [98].

A key advantage of EVs is their potential to fuse with and cross the BBB and their detectability in peripheral biofluids including blood, making them accessible and minimally invasive indicators of CNS pathology. Advances in the selective isolation of EVs populations derived from specific brain cell types have further improved the specificity of EV-based biomarkers [60,102]. Although most circulating EVs originate from peripheral tissues, a measurable fraction derives from neuronal or glial sources, enabling detection of brain-enriched proteins and RNAs in plasma. These include pathology-associated proteins such as Aβ, tau, and TDP-43, as well as disease-related RNA signatures, supporting both their diagnostic potential and their role in the intercellular propagation of pathology [103,104,105].

While EVs molecular profiling has been more extensively studied in iPSC-derived neuronal models—such as *LRRK2* mutant PD neurons, which exhibit genotype-dependent alterations in EVs cargo—similar approaches are now being applied to iPSC-derived glial systems. These studies further highlight the potential of glial EVs as cell type-specific biomarkers and mechanistic reporters of neurodegeneration [106,107,108].

Together, these findings underscore that the functional role of glial EVs in iPSC-based systems is not predetermined but is instead shaped by upstream signaling events. The same vesicle class can either propagate pathology or support neuronal resilience depending on the specific cellular and pathological environment, making the resultant effect highly context dependent (Table 2).

### 3.1. Glia-Derived EVs in AD

AD is the most common neurodegenerative disorder and a leading cause of dementia worldwide. It is neuropathologically characterized by the accumulation of extracellular β-amyloid (Aβ) plaques and intracellular neurofibrillary tangles composed of hyperphosphorylated Tau, accompanied by progressive synaptic dysfunction, neuronal loss, and chronic neuroinflammation [109].

In AD, the two most critically implicated proteins are amyloid-beta (Aβ), whose misfolding and aggregation into oligomers and plaques contribute to synaptic dysfunction and neurodegeneration and tau, a microtubule-associated protein that becomes hyperphosphorylated and forms neurofibrillary tangles, further disrupting neuronal integrity and function [110]. Beyond neuronal pathology, increasing evidence highlights the critical contribution of glial cells, particularly astrocytes and microglia, in modulating disease onset and progression [111].

Within the AD brain, pathological conditions can profoundly reshape the cargo and functional properties of ADEVs, modulating both, miRNA content such as miR-144-3p, miR-144-5p, miR-4732-5p, miR-486-5p, and miR-451 [112] as well as protein content, showing for instance soluble, β-cleaved Aβ species [113]. Astrocytic-origin EVs isolated from the plasma of AD patients, but not from Frontotemporal dementia (FTLD) or control subjects, induced complement-mediated neurotoxicity in rat cortical neurons and human iPSC-derived neurons through membrane attack complex (MAC) deposition, reducing neuronal viability by approximately 10% in MTT assays and supporting a pathogenic role for ADEVs in AD-associated complement dysregulation [99]. In particular, the accumulation of Aβ has been shown to alter ADEV composition, resulting in a reduced capacity to support neuronal survival and function [114]. Moreover, AD-associated genetic mutations may influence not only the molecular cargo packaged into EVs but also their abundance and biophysical characteristics, thereby further modulating EV-mediated communication within the neurodegenerative environment [115].

In parallel, studies using iPSC-derived microglia have revealed that MDEVs can actively participate in the dissemination of pathological proteins. When intracellular degradation pathways become inefficient, microglia can redirect misfolded proteins toward EVs secretion. Interestingly, this process appears to be selective for specific pathological conformations. For instance, pathogenic fibrillar Tau, but not its monomeric or soluble forms, has been detected in iPSC-derived MDEVs. Once released, these vesicles can enhance Tau seeding and propagation in recipient neurons, thereby facilitating the spread of pathology across neural networks [101]. These observations support the idea that MDEVs function not merely as byproducts of cellular stress but as active mediators of protein spreading under neurodegenerative conditions.

Genetic risk factors associated with AD can further exacerbate these EV-related mechanisms. Notably, microglia harboring the ApoE ε4/ε4 genotype, the strongest genetic risk factor for late-onset AD, display marked impairments in the endolysosomal system. These alterations include increased lysosomal membrane permeabilization, dysregulation of amino acid metabolism and elevated cytokine secretion. Emerging evidence suggests that such pathological changes are linked, at least in part, to modifications in EVs composition and release. Consequently, EVs pathways may represent a critical interface between microglial dysfunction, neuroinflammatory signaling and disease progression [116].

Despite the involvement of glial EVs in pathological processes, growing evidence also indicates that EVs derived from healthy stem cell-derived glial cells may exert beneficial effects. In particular, EVs produced by healthy iPSC-derived astrocytes are increasingly considered promising therapeutic candidates. Supporting this concept, Madhu and colleagues demonstrated that EVs derived from neural stem cells, when administered in a preclinical model of AD, attenuated the inflammatory cascade driven by astrocytes and microglia reducing the overall expression of inflammation related genes. Furthermore, when neural stem cell-derived EVs were applied to iMicroglia previously exposed to Aβ fibrils, the authors observed a ~20–50% reduction in the expression of microglia-specific reactive genes [94]. Importantly, their work also provided proof of principle for EVs-based therapeutic strategies by demonstrating the feasibility of intranasal EVs delivery, a minimally invasive route capable of enabling EVs access to the CNS. Together, these findings support a context-dependent role of glial EVs in AD, acting both as mediators of pathological signaling and as potential therapeutic substrates.

### 3.2. Glia-Derived EVs in PD

PD is the second most common neurodegenerative disorder and is primarily characterized by the progressive degeneration of dopaminergic neurons in the substantia nigra pars compacta, leading to motor symptoms such as bradykinesia, rigidity, and resting tremor. In addition to neuronal loss, PD is marked by the accumulation of intracellular aggregates of misfolded α-syn, known as Lewy bodies, and by significant neuroinflammatory responses involving glial cells [117]. Increasing evidence indicates that astrocytes and microglia actively contribute to PD pathogenesis through altered metabolic support, inflammatory signaling and intercellular communication mechanisms [118].

In this context, the involvement of ADEVs in PD pathology has begun to attract growing attention. While several studies have characterized ADEVs in murine models, fewer investigations have examined EVs cargo alterations in human iPSC-derived astrocytes. Nevertheless, emerging evidence suggests that disease-associated mutations can profoundly affect EVs composition and function. De Rus and colleagues demonstrated that astrocytes carrying PD-associated *LRRK2* mutations exhibit impaired EV-mediated transfer of essential neuroprotective metabolic factors. In addition their results suggest that ADEV promote the transfer of pathological phosphorylated α-syn to recipient neurons, ultimately reducing neuronal health and increasing toxicity, showed as a ~5–10% reduction in dendrite length compared to treatment with WT ADEV [56]. Consistently, studies using EVs released from iPSC-derived dopaminergic neurons carrying the *LRRK2* G2019S mutation have shown that disease-associated mutations can also alter EVs biophysical properties and molecular content. In these models, mutant cells released EVs altered protein profiles enriched in pathways linked to neurodegeneration. Importantly, correction of the *LRRK2* mutation restored EVs characteristics toward control levels, supporting the idea that EVs composition reflects the cellular disease state and may serve as a sensitive biomarker of PD-related molecular alterations [106]. In line with this concept, increased release of astrocyte-derived EVs has also been observed in the presence of α-syn deposition, likely linked to lysosomal dysfunction. Indeed, several studies have reported increased levels of α-syn-positive astrocyte-enriched EV fractions isolated from circulating biofluids of patients with PD compared with healthy controls, although methodological differences in EVs isolation and characterization currently limit cross-study comparability levels [119]. In parallel, experimental studies using iPSC-derived astrocytes have suggested that astrocytes may exert neuroprotective effects through the extracellular transfer of mitochondria or mitochondria-associated vesicles, which can be internalized by injured dopaminergic neurons in toxin-induced PD models [103]. However, the extent to which these mechanisms occur under physiological conditions or contribute to human disease in vivo remains unclear. Overall, these evidences highlight the potential value of iPSC-based models for uncovering how EV-mediated communication contributes to PD pathogenesis and for identifying potential EV-based biomarkers or new therapeutic targets.

### 3.3. Glia-Derived EVs in ALS

ALS is a progressive and fatal neurodegenerative disorder characterized by the selective degeneration of upper and lower motor neurons, leading to muscle weakness, paralysis, and ultimately respiratory failure. ALS is characterized by the great impairment of TDP-43, whose mislocalization and aggregation in motor neurons are hallmarks of the disease and contribute to neuronal dysfunction and progressive neurodegeneration [120].

Although motor neurons represent the primary cells affected, growing evidence indicates that disease progression is strongly influenced by non–cell-autonomous mechanisms involving glial cells [121].

Human induced astrocytes derived from patients carrying the *C9orf72* mutation, the most common genetic cause of familial ALS, release EVs with markedly altered miRNA cargo, including the dysregulation of more than 60 miRNAs. Among these, miR-494-3p is significantly downregulated, and EVs derived from mutant astrocytes were shown to be directly toxic to motor neurons, by inducing loss of motor neurons close to 40%. Notably, restoring miR-494-3p levels in these EVs increased motor neuron survival by approximately 25%, highlighting the functional relevance of EV-associated miRNA alterations in disease mechanisms [100]. Consistently, murine primary astrocytes overexpressing mutant *SOD1* also produce EVs that reduce motor neuron survival of almost 30% and induced a 20% reduction in neurite length, further supporting the idea that disease-associated mutations can shift astrocyte EVs signaling toward a neurotoxic phenotype [122].

Importantly, glial EVs also undergo disease-specific cargo remodeling. Proteomic analysis of EVs from iPSC-derived astrocytes obtained from a cohort of patients with ALS/Parkinsonism-dementia complex in Kii peninsula, Japan (Kii ALS/PDC), revealed increased levels of proteins related to proteostasis pathways, including unfolded protein response components, chaperones, and autophagy regulators, accompanied by reduced levels of anti-inflammatory factors [123]. Microglial EVs also appear to contribute to ALS-related inflammatory signaling. Murine microglial cells expressing the *SOD1G93A* mutation release EVs enriched in pro-inflammatory miRNAs such as miR-155 and miR-146a, which are associated with activation of NF-κB-dependent pathways. In co-culture models, EVs-mediated interactions originating from mutant glial cells lead to NF-κB activation, increased production of inflammatory cytokines, and impaired microglial phagocytic capacity, collectively amplifying neuroinflammatory responses [124].

Thus, alterations in EVs secretion and cargo, particularly dysregulated miRNAs, represent a key mechanism by which mutant glial cells influence motor neuron vulnerability in ALS. Notably, current evidence remains largely focused on astrocytes and microglia, while the contribution of EVs derived from iPSC-generated oligodendrocytes or glial progenitor cells in ALS remains largely unexplored.

## 4. Brain Organoids: A 3D Context for Disease and the Test of EVs for Therapeutic Interventions

Building on iPSC-derived models, three-dimensional (3D) brain organoids provide a more complex and physiologically system to investigate EV-mediated cell-communication. These models recapitulate key aspects of brain cytoarchitecture, enabling the analysis of EVs within a tissue-like microenvironment that more closely resembles in vivo conditions. Advances in organoid generation technologies have substantially improved the study of cell-cell communication in the brain, offering valuable insights into neurodevelopment and the mechanisms by which cellular functions are maintained, disrupted, or supported in disease contexts [125].

3D brain organoids are particularly relevant because EVs loading, release and cellular uptake are influenced by cellular density, tissue organization, maturation stage and extracellular matrix (ECM) composition. Cerebral organoids comprise a diverse array of cell types including neuroepithelial cells, radial glial cells, neuronal progenitors, neurons and astrocytes. These organoids secrete EVs with properties similar to those produced by mesenchymal stem cells (MSCs), whose therapeutic benefits have been extensively documented [126,127,128,129]. Organoid-derived EVs have been shown to reduce oxidative stress and apoptosis in midbrain astrocytes and to promote the differentiation of dopaminergic neurons, likely due to their enriched content of neurotrophic factors [130].

One of the key advantages of studying organoid-derived EVs lies in their application for evaluating neurodegeneration-related compounds. In this context, miRNA profiling has been employed to assess EVs cargo composition. Compared with traditional two-dimensional (2D) culture systems, human cerebral organoids release EVs containing miRNAs in quantities sufficient for robust profiling. Pathway analyses of these miRNAs reveal strong associations with NDs and neural signaling pathways, supporting the use of brain organoids as relevant platforms for neurotoxicity studies and early biomarker identification [131].

Thanks to their ability to generate large amounts of biological material, brain organoids are increasingly used as a human-based model to validate candidate molecules. In a recent study by Yuri Choi and collaborators, a specific EVs subpopulation positive for amyloid precursor-like protein 1 (APLP1) was identified in the blood of AD patients. To confirm their brain origin, the EV’s molecular content was validated using a brain organoid model, providing an effective alternative to direct validation in patient brain tissue, which is often limited by accessibility and would not represent early phase of the disease (where the detection of biomarkers is of greater utility) [132].

The ability to assign specific regional identities to brain organoids is a significant advantage. For example, it has been demonstrated that a particular subset of EVs, called matrix-bound nanovesicles (MBVs), are produced differently by forebrain and hindbrain organoids. MBVs are especially interesting because, although they contain relatively low amounts of microRNA, they appear to be specialized in carrying membrane proteins, including integrins, as well as lipids like glycerophospholipids and sphingolipids [133]. Although their involvement in neurodegeneration has not been fully understood yet, MBVs have shown neuroprotective potential by shifting microglia and astrocytes toward anti-inflammatory phenotypes. They can suppress pro-inflammatory cytokine release and increase retinal ganglion cell viability from 0% in neurotoxic conditions to 126% of control, either by directly supporting neurons or neutralizing toxic astrocyte-derived signals in a model of ischemia-induced retinal ganglion cell axon degeneration [134].

Together, iPSC-derived glial cells and brain organoids bridge the gap between conventional in vitro systems and in vivo studies, providing dynamic and scalable platforms for investigating the biogenesis, cargo, and functional impact of EVs in health and disease. These advances may contribute to the development and evaluation of EV-based diagnostic and therapeutic strategies for neurological disorders.

## 5. Current Limitations and Challenges in EVs Research

Despite the growing interest in EVs as biomarkers and therapeutic tools, several important limitations and unresolved challenges still remain. A major issue in the field is the pronounced heterogeneity of EVs populations, which complicates the identification of functionally distinct vesicle subtypes and limits comparability across studies [135,136]. Current isolation methods frequently co-purify contaminants such as lipoproteins, protein aggregates, ribonucleoprotein complexes, and non-vesicular particles, particularly when analyzing complex biofluids including plasma and cerebrospinal fluid [62,137]. Consequently, differences in EVs isolation, purification, and characterization protocols can substantially affect EVs yield, cargo composition, and downstream functional interpretation. In several cases, studies using different isolation workflows have reported conflicting findings regarding EVs abundance, molecular signatures, and biological activity, highlighting persistent reproducibility challenges across laboratories [138,139,140].

In addition, EVs cargo composition is highly dynamic and strongly influenced by donor variability, cell culture conditions, cellular activation states and environmental stressors, making it difficult to distinguish disease-specific alterations from experimental variability. Although numerous preclinical studies suggest neuroprotective or regenerative effects of EVs, their therapeutic efficacy has not been sufficiently proven, and robust validation in vivo and in clinical implementation is still limited. Moreover, important translational barriers also remain unresolved, including large-scale production, long-term storage stability, biodistribution, targeting specificity, immunogenicity and batch-to-batch consistency [64,141,142,143,144]. Together, these limitations underscore that EVs should not be considered universal therapeutic agents, but rather complex and context-dependent biological mediators whose clinical utility will require rigorous standardization, mechanistic validation and careful interpretation.

## 6. Conclusions and Future Directions

Although glial-derived EVs are increasingly implicated in neurodegenerative diseases, their biological effects appear highly dependent on cellular and pathological context. Current evidence suggests that EV function is influenced by glial subtype, activation state, intracellular degradative capacity, disease stage and cargo composition. Under physiological conditions, EVs released by homeostatic astrocytes, microglia, and oligodendrocytes generally support neuronal survival, synaptic function, and tissue homeostasis through the transfer of neurotrophic factors, regulatory miRNAs and metabolic components. In contrast, under inflammatory or neurodegenerative conditions, reactive glial cells may release EVs enriched in misfolded proteins, pro-inflammatory mediators and stress-associated cargo, thereby contributing to neurotoxicity and disease propagation. Importantly, lysosomal dysfunction and impaired autophagy may further promote the extracellular release of toxic material. However, EV secretion may also represent a compensatory response aimed at maintaining intracellular proteostasis. Together, these findings highlight that glial EVs cannot be considered uniformly protective or pathogenic but rather must be interpreted within specific cellular and disease contexts.

EVs possess several properties that have stimulated interest in their potential therapeutic application, including relative biocompatibility and the capacity, in certain experimental contexts, to deliver bioactive cargo across or in association with the BBB. However, EVs biodistribution and BBB permeability appear to be strongly dependent on EVs origin, molecular composition, route of administration, disease state, and engineering strategy. Importantly, the mechanisms and efficiency of EVs transport across the BBB remain incompletely understood and current evidence varies substantially depending on experimental models, EVs isolation methods, labeling approaches and administration routes.

Glia-derived EVs are emerging as critical mediators of intercellular communication in the CNS, influencing both physiological and pathological processes (Figure 3). Given the limitations of current treatments for NDs, EVs are increasingly being investigated as potential therapeutic tools because of their role in intercellular communication and their capacity to transport diverse bioactive molecules.

The advent of iPSCs has significantly boosted the study of glial EVs by providing scalable, genetically tractable and patient-specific platforms to model NDs [17,145]. iPSC-derived glial cells, including astrocytes, oligodendrocytes and microglia recapitulate key human-specific and disease-relevant phenotypes, enabling the investigation of EVs biogenesis, cargo specificity and functional effects in a human-relevant context. These systems also facilitate the study of glia–neuron interactions and support biomarker discovery and translational research [102].

Nonetheless, several challenges remain. Indeed, differentiation protocols are often time-consuming and variable across laboratories, contributing to heterogeneity in EVs populations [146,147,148,149,150,151,152,153]. Thus, while in vitro systems cannot fully recapitulate the complexity of the human CNS, increasingly sophisticated brain organoid models partially address this limitation. However, the lack of fully developed vascularization and immune components, together with organoid-to-organoid variability, still constrain their physiological relevance. Complementary in vivo validation using humanized or chimeric models will therefore be essential.

**Figure 4:**
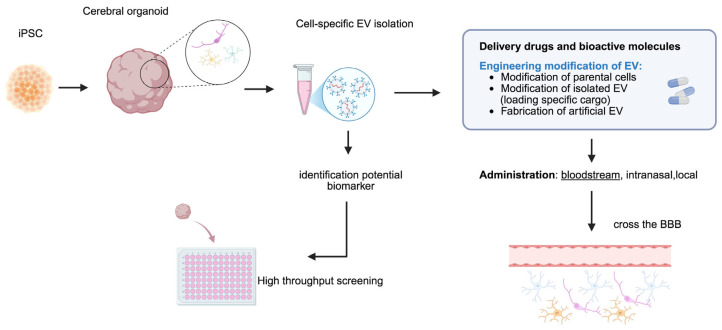
iPSC-derived EVs as therapeutics and disease signature sources. iPSC-derived neural cells serve as a robust source of EVs. iPSCs can be differentiated into brain organoids that partially reproduce the complexity of the 3D brain environment, including multiple neural cell types. This enables the isolation of cell-type specific EVs, which can be used either to identify disease-relevant molecular signatures (biomarkers), assessable through high-throughput screening, or to detect biocompatible therapeutic molecules which cross the BBB. EVs can also be engineered by various approaches (highlighted in the blue box) and have the potential to cross the BBB, positioning them as promising vectors for drug delivery. This figure was created using BioRender: https://BioRender.com/gau7fmc, accessed on 24 May 2026.

Importantly, donor-dependent variability in iPSC models, rather than representing a limitation, reflects genuine inter-individual genetic differences. This provides a unique opportunity to distinguish shared disease signatures from patient-specific EVs profiles, supporting the development of precision medicine approaches. In parallel, iPSC-derived systems are increasingly used for high-throughput screening and validation of therapeutic candidates, and their scalability opens the possibility for large production of EVs as therapeutic agents (Figure 4).

EVs possess several intrinsic features that make them attractive therapeutic vectors, including biocompatibility, the ability to cross the BBB and the capacity to deliver diverse molecular cargos [82,150]. Advances in EVs isolation, engineering and targeting strategies have further enhanced their therapeutic potential, enabling improved cargo loading, stability and cell-type specificity [151,152]. Engineering approaches—such as genetic modification of parental iPSCs or surface functionalization of EVs—are transforming these vesicles into programmable delivery systems for nucleic acids, proteins and small molecules.

Recent studies highlight the translational potential of engineered iPSC-derived EVs. For example, brain-targeting EVs generated through RVG-Lamp2B knock-in strategies exhibit improved CNS delivery, while engineered vesicles such as “tentacled” EVs demonstrate the feasibility of active cargo transport and functional recovery in disease models [153,154]. However, therapeutic applications remain more advanced in AD than in PD or ALS, highlighting an important needed for future research.

A major unresolved question concerns how EVs cargo reflects disease stage. iPSC-based models provide a powerful platform to address this question, although they lack age-associated features due to cellular reprogramming. Emerging strategies-including prolonged maturation, induction of cellular aging, multicellular co-culture systems and controlled stress paradigms-offer ways to model disease progression. Longitudinal EVs profiling in these systems may reveal early molecular signatures preceding neurodegeneration, facilitating the identification of stage-specific biomarkers and therapeutic targets.

Looking ahead, the integration of EVs profiling with patient-specific iPSCs models is expected to play a central role in precision medicine, enabling improved disease stratification, monitoring and therapeutic intervention. Continued interdisciplinary efforts combining stem cell biology, bioengineering and clinical research will be essential to fully exploit the potential of glial EVs. Nevertheless, important challenges remain, including the heterogeneity of EVs populations, limited standardization of isolation procedures, variability among iPSC-derived models, and the need for robust in vivo and clinical validation. Addressing these limitations will be essential before EVs-based approaches can be reliably translated into clinical applications. In conclusion, although significant progress has been made, key outstanding questions remain (see Box 1), providing important directions for future research. Together, these findings support the use of iPSC-derived glial models as valuable experimental platforms for biomarker discovery and for investigating candidate therapeutic strategies in neurodegenerative disorders.

Box 1 **Key Outstanding Questions.**

- How is EVs cargo selectively regulated in different glial cell types under physiological versus pathological conditions?
- To what extent do glial-derived EVs actively drive disease progression versus reflect ongoing pathology?
- How does genetic background influence glial EVs composition and function?
- How well do iPSC-derived and organoid-based models recapitulate EVs dynamics observed in the human brain in vivo?
- Can engineered or endogenous glial EVs be safely and efficiently harnessed as therapeutic delivery systems in neurodegenerative diseases?

## Figures and Tables

**Table 1:** Summary of EV-based clinical trials in brain disease.

NCT Number	Clinical Indication	Study Type	Diagnostic EVs Sources	Readout
NCT05807581	Parkinson’s Disease	Biomarkers and effectiveness of therapies	CSF	Tau (t-Tau), neurofilament light chain, (NfL), and phosphorylated neurofilament heavy chain, (p-NfH), synaptic dysfunction α-synuclein and neurogranin, (Ng), neuroinflammation
NCT05832255	Fragile X Syndrome	Effectiveness of treatmen	Buccal swab and saliva	Serotonin levels in exosomes
NCT05838573	Schizophrenia	Biomarkers	Blood	Changes of level of phosphorylated insulin receptor substrate 1 and its downstream mediators in Extracellular Vesicles of neuronal origin isolated from blood
NCT05843552	Gaucher Disease	Correlation with disease	Plasma	Quantity, size, content
NCT05886205	Refractory Focal Epilepsy	safety, tolerability, and preliminary efficacy		safety, tolerability, and preliminary efficacy of GD-iEXo-002 nasal drop
NCT05902065	Parkinson’s Disease	Correlation of factors with rehabilitation after treatment	Blood	Raman spectra of EVs before and after treatment
NCT05913960	Depressive Disorder	Association with effectiveness of treatment	Blood	Protein content over time
NCT05915312	Bipolar Affective Disorder	Accuracy at predicting BD versus MDD or healthy	Blood	microRNA and related proteins in exosomes
NCT05927129	Depression Based on the fNIRS	Effectiveness of treatment and associated change	Blood	Factors carried by blood, neurotrophic factor, reelin
NCT05977088	Alzheimer’s Patients	Effectivness of Treatment	Blood	ELIA kit of blood EVs
NCT06082713	Huntington Disease	Biomarker study	Blood	Expression of proteins or specifically Huntingtin protein in brain-derived EVs in human biofluids from HD patients as compared to non-HD patients
NCT01716481	Ischemic Stroke	Biomarker study	Blood	Categorical shift in modified Rankin scale (mRS)

**Table 2:** Summary of cell-type specific EV cargo in healthy and diseased conditions.

Cell Types	Origin Cells	Disease/Context	Molecule Secreted (EV Cargo)	Effect	Ref.
Astrocytes	Murine	Healthy CNS	BDNF, GDNF, NGF	Promote neuronal survival and synaptic function	[75]
Astrocytes	Murine	Healthy CNS vs. Fragile X Syndrome	Lactate, mitochondrial support factors	Maintain neuronal metabolism and energy homeostasis	[77]
Astrocytes	Human astrocytes from brain tissue and human primary astrocytes	Healthy CNS	Heat shock proteins, LRP1, KCTD12, G6PD, KIF5B, SPTAN1	Promote neuronal differentiation and electrophysiological maturation	[78,79]
Astrocytes	iPSC- derived astrocytes (44) and mouse astrocytes (48)	Healthy CNS	miR-21, miR-29a, miR-124, miR-26a	Regulate neuronal gene expression, synaptic plasticity, and neurogenesis	[80,84]
Astrocytes	iPSC- derived astrocytes	Healthy CNS	VEGF, FGF2, ANGPT1, miR-126	Promote angiogenesis and maintain BBB integrity	[85]
Astrocytes	iPSC-derived astrocytes	Alzheimer’s disease	Altered EV cargo after Aβ exposure	Reduced neuronal support and neuroprotection	[99]
Astrocytes	iPSC- derived astrocytes	Parkinson’s disease	α-synuclein, reduced metabolic factors	Increase neuronal toxicity and impair dopaminergic neuron survival	[56]
Astrocytes	iAstrocyes	ALS (C9orf72 mutation)	Reduced miR-494-3p, Reduced miR-17~92 cluster, miR-29 family	Impaired neuronal support and synaptic stability	[100]
Microglia	iPSC- derived astrocytes	Stroke/neuroinflammation	TMEM119^+^/CD14^+^, TMEM119^+^/MHC-II^+^ EV markers	Reflect microglial activation and inflammatory states	[84]
Microglia	iPSC-derived microglia	Alzheimer’s disease	Fibrillar Tau	Promote Tau seeding and propagation in neurons	[101]
Microglia	Murine	Alzheimer’s disease	Neuroprotective miRNAs and proteins	Reduce amyloid burden, decrease neuroinflammation, improve cognition	[94]
Oligodendrocytes	Murine	Healthy CNS	MBP, PLP, MOG, Sphingolipids, Cholesterol, Lactate dehydrogenase, miR-219, miR-338	Support axonal stability and myelin maintenance	[90]

## Data Availability

No new data were generated in this study. Data sharing is not applicable to this article.

## Abbreviations

AD	Alzheimer disease
APLP1	Amyloid precursor-like protein 1
ALS	Amyotrophic lateral sclerosis
ADEVs	Astrocytes-derived extracellular vehicles
BBB	Blood-brain barrier
CNS	Central nervous system
DGUC	Density-gradient ultracentrifugation
ESCRT	Endosomal Sorting Complex Required for Transport
EVs	Extracellular vesicles
FTLD	Frontotemporal dementia
G6PD	Glucose-6-phosphate dehydrogenase
iPSCs	Induced pluripotent stem cell
ILVs	Intraluminal vesicles
KIF5B	Kinesin family member 5B
LRP1	Lipoprotein receptor-related protein 1
MBVs	Matrix-bound nanovesicles
MAC	Membrane attack complex
MSCs	Mesenchymal stem cells
mRNA	Messenger RNA
MDEVs	Microglia-derived extracellular vesicles
miRNAs	MicroRNAs
MBP	Myelin-associated proteins such as myelin basic protein
MOG	Myelin oligodendrocyte glycoprotein
MVBs	Multivesicular bodies
NTA	Nanoparticle tracking analysis
ND	Neurodegenerative disease
ODEVs	Oligodendrocyte-derived extracellular vehicles
PD	Parkinson disease
KCTD12	Potassium channel tetramerization domain-containing 12
PLP	Proteolipid protein
rRNA	Ribosomal RNA
SEC	Size-exclusion chromatography
SPTAN1	Spectrin-alpha non-erythrocytic 1
TFF	Tangential flow filtration
DUC	Ultracentrifugation
